# Measurement of early bone loss around an uncemented femoral stem: A methodological study with dual-energy X-ray absorptiometry in 16 patients

**DOI:** 10.3109/17453674.2011.579519

**Published:** 2011-07-08

**Authors:** Berte Bøe, Tore Heier, Lars Nordsletten

**Affiliations:** ^1^Department of Orthopaedics, Oslo University Hospital, Ullevål; ^2^Faculty of Medicine, University of Oslo; ^3^Surgical Department, Diakonhjemmet Hospital, Oslo, Norway

## Abstract

**Background and purpose:**

Dual-energy X-ray absorptiometry (DXA) is a precise method to study changes in bone mineral density (BMD), including the pattern of bone remodeling around an implant. Results from implant studies are usually presented as changes in BMD as a function of time. The baseline and reference value for such calculations is the first measurement after the operation. The baseline measurement has been performed at different time points in different studies. If there is rapid bone loss immediately after an operation, this will influence the reference value and hence the results. To evaluate DXA as a method, we studied the very early changes by doing 3 DXA measurements within the first 2 weeks after surgery.

**Patients and methods:**

We included 23 hips in 23 patients who were operated with an uncemented total hip prosthesis (THP). Each Gruen region was measured with DXA at 1, 5, and 14 days, and 3 and 12 months after the operation. 16 of the patients completed all 5 follow-ups.

**Results:**

There was no detectable change in BMD in the first 14 days after the operation. In all zones, the lowest BMD was measured after 3 months.

**Interpretation:**

We conclude that DXA measurements done within 14 days after the operation can be used as reference measurements for later follow-up studies.

The bone remodeling around hip prostheses appears to vary a great deal with different fixation methods and stem designs (Kiratli et al. 1996, Boden et al. 2004, Rahmy et al. 2004, Grant et al. 2005). Even with the same implant, researchers have reported a variety of bone mineral density (BMD) changes. In implant research, BMD results are most often given in percentage change relative to the first postoperative measurement. The postoperative measurement is used as a reference to avoid measuring the changes in BMD due to the operation (Kroger et al. 1996). During surgery, bone is removed and compacted due to rasping and insertion of the stem. The reference measurement is of importance because it influences all later results. Aamodt (2004) presented 2-year dual-energy X-ray absorptiometry (DXA) results, with 23% bone loss in Gruen zone 7 for the ABG-1 stem. Van der Wal et al. (2008) reported 2 patient groups with 12% and 15% reduction in BMD in zone 7 for the same femoral stem. The only obvious difference in these 2 studies was the timing of the first measurement. Van der Wal performed the baseline measurement at 10 days postoperatively while Aamodt performed the first postoperative measurement 3–5 days after the operation. Rapid bone loss from day 3–5 to day 10 could therefore have explained the difference in bone loss at 2 years.

It is not fully known whether the bone loss starts immediately after the operation or after a few weeks. In the early postoperative period, BMD might change because of disuse atrophy (McCarthy et al. 1991) or because of the trauma to the bone (Karlsson et al. 2000). We hypothesized there is a rapid bone loss in the first days after operation, which would be an important source of bias to postoperative reference measurements.

## Patients and methods

We included 23 patients (15 women) who were operated with an uncemented HA-coated Corail stem (DePuy International Ltd., Leeds, UK). The inclusion criterion was indication for THA with an uncemented stem. Exclusion criteria were infection, revision arthroplasty, marked bone loss, medication with bone-active drugs, or severe morbidity. Mean age at time of operation was 64 (34–82) years. Recruitment was through informed consent. The Norwegian Data Inspectorate and the regional ethics committee approved the study, and it was carried out according to the Helsinki declaration.

BMD was measured with DXA by experienced technicians. 2 different DXA machines were used in 2 different institutions (Prodigy; Lunar, Madison, WI and Hologic QDR; Hologic Inc., Bedford, MA). Each patient was measured on the same machine on all occasions. The patients were placed supine on the scan table with a foot support to achieve a standard rotation of the hip. Orthopaedic software (Lunar version 1.2 and Hologic QDR version 12.3) was used to analyze periprosthetic BMD in 7 regions of interest (ROIs). The ROIs were based on the Gruen zones. The patients were measured 1–2 days postoperatively, and on days 5 and 14. For follow-up, they were measured after 3 months and 1 year. 16 patients underwent all 5 measurements.

### Statistics

The results were calculated as change in percent. Differences were compared by Wilcoxon signed rank test (non-parametric) using PASW statistics software version 18.0 (SPSS). To calculate precision error, all examinations were repeated on the same day with repositioning between the scans. The differences between these paired BMD measurements were used to calculate the coefficient of variation (CV) for each ROI: CV% = 100 × [(δ/√2)/μ] for each ROI, where δ represents the standard deviation of the difference between the paired BMD measurements, and μ is the overall mean of all the BMD measurements for each individual ROI.

## Results

The precision (CV) of DXA measurements varied from 0.8% in Gruen zone 4 to 5.1% in zone 7. Mean CV was 1.8% (Table 1).

**Table 1.** The coefficient of variation (CV%) of the BMD measurements in different Gruen zones and overall

	Zone 1	Zone 2	Zone 3	Zone 4	Zone 5	Zone 6	Zone 7	Overall
1 day	1.5	3.0	3.4	0.8	2.2	0.9	1.0	2.1
5 days	1.2	2.9	1.9	1.1	2.6	1.7	2.0	2.4
14 days	0.9	1.7	1.3	0.7	0.7	0.9	1.8	1.7
3 months	1.2	1.0	1.1	1.0	1.0	1.1	1.0	1.4
12 months	2.1	1.8	1.9	3.1	3.0	2.7	5.1	2.0

**Figure 1.**
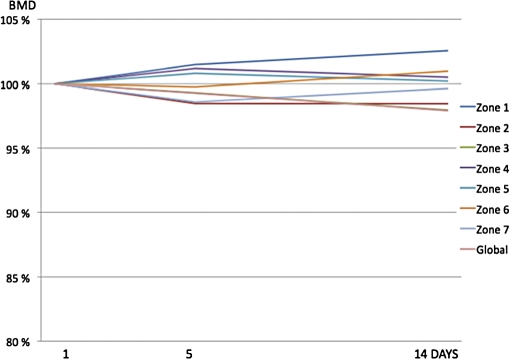
Changes in bone mineral density (BMD) in different regions over the first 14 days after insertion of an uncemented femoral stem. Results are medians, given as percentages of the first postoperative value.

**Figure 2.**
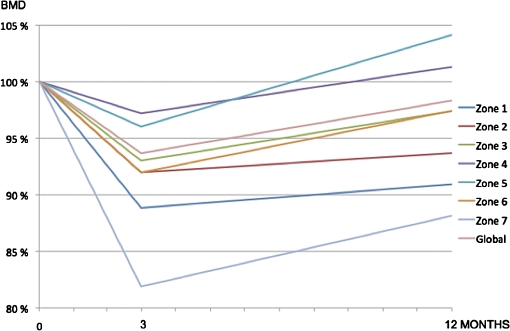
Changes in bone mineral density (BMD) in different regions during the first year after insertion of an uncemented femoral stem. Results are medians, given as percentages of the first postoperative value.

There was no change in BMD in the first 2 weeks postoperatively (Figure 1). Between 14 days and 3 months, there was a mean bone loss of 8%, ranging from 18% in zone 7 to 4% in zones 4 and 5 (p < 0.05 for all zones). There was restoration of bone in all zones from the 3-month to the 12-month follow-up (Figure 2). This restoration was from 2% to 8% and was statistically significant in zones 2–6 (p = 0.02, p < 0.001, p < 0.001, p = 0.001, and p = 0.05). The BMD decreased most in zone 7: by 18% after 3 months (Table 2).

**Table 2.** Median bone mineral density in different regions after insertion of an uncemented femoral stem. Results are percent (range) after 5 days, 14 days, 3 months, and 12 months

	Zone 1	Zone 2	Zone 3	Zone 4	Zone 5	Zone 6	Zone 7	Global
5 days	101 (95–110)	98 (92–101)	99 (89–122)	101 (98–107)	101 (91–108)	100 (91–117)	99 (89–113)	99 (95–120)
14 days	103 (96–107)	98 (90–106)	98 (90–125)	101 (96–112)	100 (91–107)	101 (92–109)	100 (91–109)	98 (90–111)
3 months	89 (77–109)	89 (74–103)	93 (80–137)	97 (94–107)	96 (82–104)	92 (79–120)	82 (70–101)	94 (88–110)
12 months	91 (73–102)	94 (84–111)	97 (90–139)	101 (95–114)	104 (93–127)	97 (82–114)	88 (66–100)	98 (91–114)

## Discussion

We found similar BMD values during the first 14 days after implantation of an uncemented femoral stem. Baseline measurement for bone remodeling studies with DXA can therefore be done at any time in this interval.

Bone changes around implants have been an area of continued interest. Aseptic loosening is thought to be the consequence of bone loss (often attributed to stress shielding) and an inflammatory process induced by foreign body particles (Bobyn et al. 1992, Van Rietbergen et al. 1993, Hoenders et al. 2008). The most informative and frequently used way to present periprosthetic bone remodeling is relative change as a function of time. Since there are different forms of bias related to use of preoperative measurements, such as disuse atrophy, sclerotic bone and peroperative bone loss, the recommended baseline is the first postoperative measurement (Venesmaa et al. 2001). Van der Wal et al. (2008) stated that studies comparing BMD after operation with THA should be matched for preoperative BMD and sex. A correlation between preoperative BMD and postoperative bone loss indicates that the lower the BMD before operation, the higher is the bone loss after the operation (Venesmaa et al. 2003, Rahmy et al. 2004, Alm et al. 2009). Kiratli et al. (1996) defined 4 ROIs in the proximal femur of the operated hip and did not find any correlation between preoperative BMD and the postoperative bone-remodeling pattern. Their first postoperative measurement, done less than 5 days after the operation, showed an increased BMD in all 4 ROIs. All values almost returned to baseline within 1 month.

The most marked postoperative bone loss takes place in the first months after the operation. Bone loss of up to 21% has been reported in the first 3 months in Gruen zones 1 and 7 (Boden and Adolphson 2004). As in our study, Trevisan et al. (1997) also found an increase in BMD in the greater trochanter early after the operation. They took the first postoperative measurements at 2 months and compared these values with preoperative values. BMD appears to stabilize after approximately 6 months (Kroger et al. 1997). The main reason for bone loss is thought to be partial weight bearing and stress shielding. Boden and Adolphson (2004) compared BMD in 20 patients randomized to partial or full weight bearing. The group with partial weight bearing had lost more bone in Gruen zones 1, 4, and 5 after 3 months than those with full weight bearing. Differences in BMD between study groups appear to level off with time. In the material of Boden and Adolphson, the difference due to weight bearing only remained for 2 years in zone 1. Thien et al. (2010) compared a polymethyl methacrylate-coated (PMMA-coated) stem, a polished stem, and a matte stem. Initially, the polished stem lost less bone and subsided more then the other two. After 5 years of follow–up, there was no difference. In our own study on Taperloc stems (Bøe et al. 2011), there was a significant difference in bone remodeling in the major trochanter between 2 different hydroxyapatite-coated stems during the first 2 years.

In addition to having reliable reference measurement, longitudinal studies depend on reproducibility. Rotation of the femur appears to be the most significant factor affecting reproducibility (Cohen and Rushton 1995). Repositioning of the patient between 2 measurements on the same day should give us reliable feedback on the precision of DXA measurements. In our study, a coefficient of variation below 5% indicated good precision, and daily scanning of a phantom allowed us to check for drift of the DXA machines.

Another factor influencing BMD around implants is operation technique. Compaction is a bone-saving technique compared to conventional bone-removing techniques by a rasp with similar shape as the stem. Using a dog model, Kold et al. (2005a) showed that operation with compaction around an HA-coated implant increased the peri-implant bone density and bone implant contact. This indicates that compaction may be an advancement in human joint replacement to enhance initial fixation. Even though compaction represents autograft of non-vital bone, which is resorbed over time, the fixation does not appear to be inferior after bone resorption (Kold et al. 2005b). To our knowledge, the technique has not been tested in a human clinical trial. Perhaps compaction is the reason for increased BMD immediately after surgery, since most of the rasps that are used can probably both compact and remove bone. It is of some concern that the risk of femoral fractures increases with compaction (Kold et al. 2003).

Bone remodeling after implantation of prostheses may be compared to remodeling after partial weight bearing because of fractures. Eyres and Kanis (1995) found a definite and persistent loss of BMD in the distal tibia 5–11 years after fracture. At the fracture site, there was sclerosis and a higher BMD than on the control side. In that material, there was no improvement in BMD with weight bearing. Fractures sustained in childhood did not lead to bone loss in the distal tibia. There have been several publications indicating that bone loss may be the result of a fracture, and not necessary the cause of it (Andersson and Nilsson 1979, Eyres and Kanis 1995, Karlsson et al. 2000). Karlsson et al. (2000) published BMD results from both legs, both hips, spine, and total body in patients who were operated with tibial osteotomy for localized medial osteoarthritis. They found substantial bone loss in the whole body, the spine, and the contralateral hip after 9 and 15 months. In the leg with osteotomy, the bone loss was significant in the distal femur after 4 months (compared to baseline) and in the shaft of the tibia after 9 and 15 months. The conclusion from that work was that bone loss following an osteotomy is rapid, affects both fractured and unfractured bones, and is not completely reversible. The same mechanisms may be responsible for the bone remodeling seen after implantation of prostheses, but the exact reason for this “post-traumatic” bone loss is unknown.
